# Preoperative antibiotic prophylaxis in primary shoulder arthroplasty patients: a systematic review

**DOI:** 10.1016/j.jseint.2025.06.010

**Published:** 2025-07-07

**Authors:** Kevin C. Liu, Justin T. Jabara, Miguel A. Lizarraga, Andrew P. Gatto, Brian Feeley

**Affiliations:** Department of Orthopaedic Surgery, University of California, San Francisco, San Francisco, CA, USA

**Keywords:** Shoulder arthroplasty, Antibiotic prophylaxis, Cefazolin, Vancomycin, Clindamycin, Doxycycline

## Abstract

**Background:**

Cefazolin is the primary antibiotic used for prevention of shoulder arthroplasty (SA) infection. However, vancomycin, clindamycin, and doxycycline may be used, specifically for patients with penicillin allergies or known bacterial colonization. Due to limited existing data, the aim of this systematic review was to characterize contemporary antibiotic prophylaxis choices and report infection rates based on the prophylactic regimen in patients undergoing SA.

**Methods:**

The online databases CINAHL Complete, EMBASE, MEDLINE, the Cochrane Central Registry of Controlled Trials, and Web of Science were searched from database inception to September 25, 2024. Clinical studies comparing preoperative antibiotic regimens and reporting postoperative complications were included. Nonrandomized and randomized studies were assessed using the Methodological Index for Non-Randomized Studies tool and the revised Cochrane Risk of Bias 2 tool, respectively.

**Results:**

The search strategy identified 7 eligible studies, of which 2 were randomized controlled trials and 5 were retrospective series, including 33,159 procedures (hemiarthroplasty, anatomic total shoulder arthroplasty, reverse total shoulder arthroplasty, and humeral head arthroplasty). The most commonly used antibiotic was cefazolin. All-cause infection rates ranged from 1.0%-1.1% for cefazolin, 1.1%-2.4% for vancomycin, and 3.2%-4.1% for clindamycin. One randomized controlled trial found no significant reduction in intraoperative culture positivity rates with the addition of doxycycline to cefazolin.

**Conclusion:**

Cefazolin is the preferred antibiotic prophylaxis for SA, with vancomycin and clindamycin as viable alternatives. Future investigations could evaluate the benefit of dual antibiotic therapy and develop evidence-based treatment algorithms for high-risk patients who may require non–cefazolin prophylaxis.

The prevalence of shoulder arthroplasty (SA) has increased significantly over the past 2 decades, with a prevalence of over 800,000 procedures in 2017 and projected 4-fold increase by 2030.^7^^,^^21^ One of the most challenging complications associated with SA is periprosthetic joint infection (PJI). Optimization of antibacterial control strategies, particularly to prevent *Cutibacterium acnes (C. acnes)* infection, is critical. Several options have come into favor, including various sterile skin preparations,^17^ the use of electrocautery during dermal dissection,^11^ and specific antibiotic prophylactic protocols.^12^

Preoperative antibiotic prophylaxis aims to reduce bacterial burden in the operative shoulder before prosthesis implantation. The most common bacterial culprit in SA is *C. acnes*, followed by *Staphylococcus aureus* (*S. aureus*).^9^ Despite an understanding of the common microbiology associated with PJI in SA, the problem persists. The majority of patients undergoing SA receive preoperative cefazolin, which has demonstrated efficacy in PJI prevention due to its broad coverage and bactericidal mechanism.^1^ However, in patients with a reported penicillin or cephalosporin allergy, or known methicillin-resistant *Staphylococcus aureus* (MRSA) colonization, the preoperative team often must decide whether to utilize cefazolin or consider alternative antibiotics. The most commonly employed alternative antibiotics include vancomycin, clindamycin, and doxycycline.^3^ Whether these alternatives have equal efficacy or leave patients more susceptible to PJI is an area of ongoing research.

Cefazolin has been extensively studied in the context of joint arthroplasty, particularly in hip and knee surgery.^31^ However, due to the overall rarity of true penicillin/cephalosporin allergy, there is limited data comparing cefazolin to non–cefazolin prophylactic regimens in SA patients.^27^^,^^30^^,^^33^ Therefore, the objective of this systematic review was to assess the current body of literature surrounding SA antibiotic prophylaxis, to characterize contemporary antibiotic prophylaxis choices, and to report infection rates based prophylactic regimen in patients undergoing SA.

## Materials and methods

This systematic review was performed in accordance with the Preferred Reporting Items for Systematic Reviews and Meta-Analyses guidelines.^18^

### Eligibility criteria

We included clinical studies (randomized clinical trials, case-control studies, and prospective or retrospective cohort studies) in which patients underwent anatomic total shoulder arthroplasty, reverse total shoulder arthroplasty, hemiarthroplasty, or humeral head resurfacing; a preoperative intravenous antibiotic prophylactic regimen was compared with any other preoperative intravenous regimen or with no antibiotic prophylaxis; and the incidence of postoperative infection or culture positivity was reported. Only studies published in English were included. We excluded studies involving revision shoulder arthroplasty or patients with known prior infections. Only primary research was considered for review.

### Data sources and search strategy

**Figure 1:**
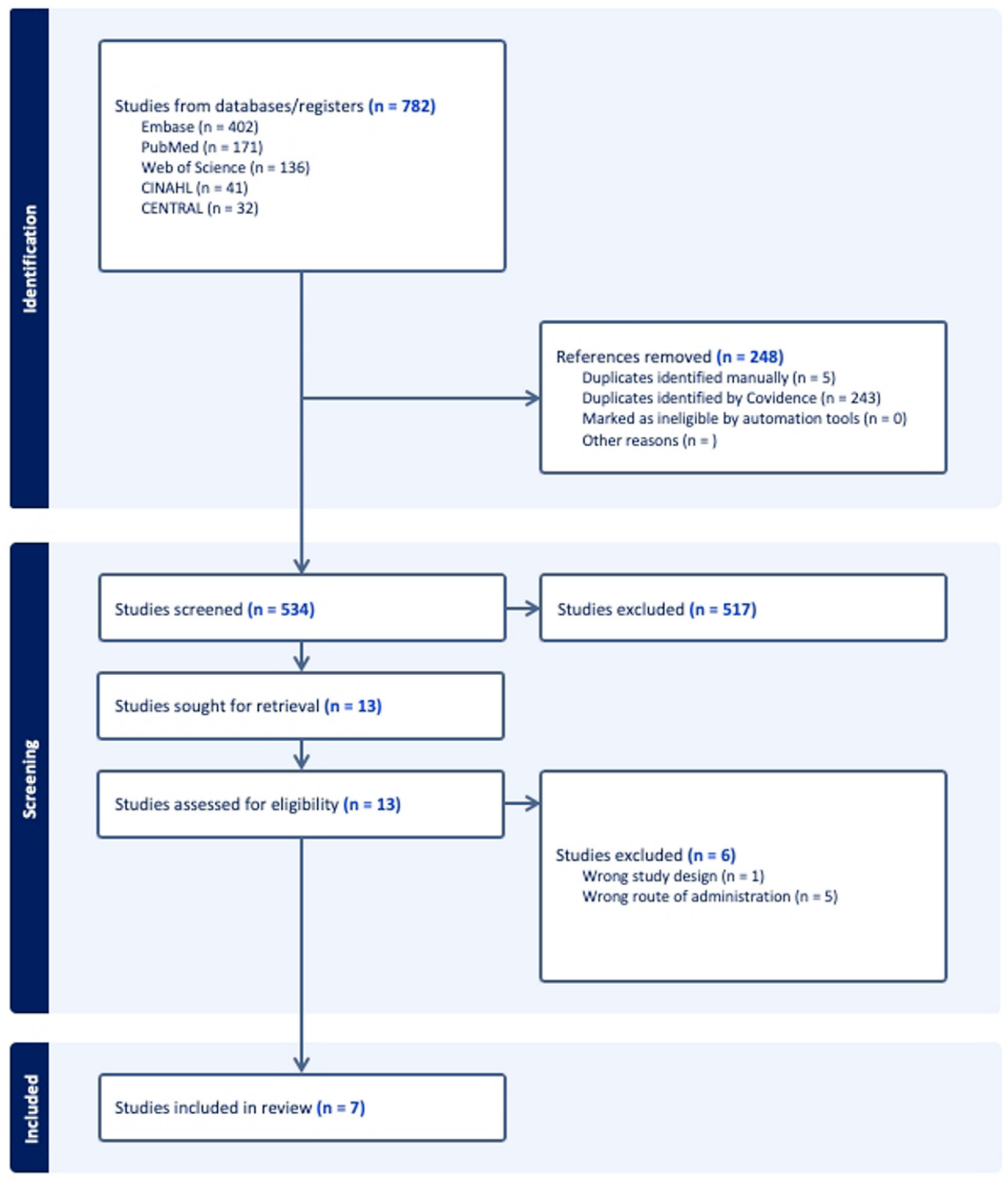
Flowchart of systematic review process.

The searches for this review were performed on September 25, 2024. The CINAHL Complete, EMBASE, MEDLINE, the Cochrane Central Registry of Controlled Trials, Web of Science databases were searched from database inception to September 25, 2024. A thorough manual examination of the reference lists in the identified articles was carried out to find any potentially relevant studies. Our search strategy is available in Figure 1, and exact search terms can be found in the Appendix.

Title and abstract screening, review of full texts for potential studies, and eligibility assessments were conducted independently by 2 reviewers (KCL and MAL). If disagreements were encountered, a third independent reviewer (JTJ) assisted with resolution. The data extracted from the included studies included several variables, such as publication year, study design, participant number, mean age, sex, presence of methicillin-resistant bacterial colonization, presence of penicillin allergy, infection rates, and culture positivity rates.

### Assessment of risk of bias

Risk of bias of the included studies were evaluated by 2 authors (KCL and MAL) using validated tools previously used in the SA literature.^10^ Nonrandomized studies were assessed with the Methodological Index for Non-Randomized Studies tool (Table I). The Methodological Index for Non-Randomized Studies system evaluates the methodological quality of nonrandomized studies using a 12-item index, scored from 0 (not reported) to 2 (adequately reported).^28^ Randomized trials were assessed using the revised Cochrane Risk of Bias 2 tool (Table II). The Risk of Bias 2 tool evaluates the risk of bias across 5 domains: randomization process, deviations from intended interventions, missing outcome data, outcome measurement, and selection of the reported result.^29^ Discrepancies in quality assessment were resolved with a third author (JTJ).

**Table I:** MINORS scores for nonrandomized studies.

Study	Dettmer et al (2023)^6^	Marigi et al (2022)^13^	Marigi et al (2023)^14^	Marigi et al (2024)^15^	Yian et al (2020)^32^
A clearly stated aim	2	2	2	2	2
Inclusion of consecutive patients	2	2	2	2	2
Prospective collection of data	2	2	2	2	2
Endpoints appropriate to the aim	2	2	2	2	2
Unbiased assessment of study endpoint	0	0	0	0	0
Follow-up period appropriate	0	2	2	2	2
Loss to follow-up less than 5%	0	2	2	2	2
Prospective calculation of study size	0	0	0	0	0
Adequate control group	2	2	2	2	2
Contemporary groups	2	2	2	2	2
Baseline equivalence of groups	0	1	1	1	1
Adequate statistical analyses	1	2	2	2	2
Total score	13	19	19	19	19

*MINORS*, methodological index for non-randomized studies.

0: not reported; 1: reported by inadequate; 2: reported and adequate. The global ideal score is 16 for noncomparative studies and 24 for comparative studies.

**Table II:** ROB 2 scores for randomized trials.

Study	Rao et al (2018)^25^	Peel et al (2023)^24^
Bias from randomization process	Low risk	Low risk
Bias from deviations from intended interventions	Low risk	Low risk
Bias due to missing outcome data	Low risk	Low risk
Bias in measurement of the outcome	Some concerns	Low risk
Bias in selection of the reported result	Low risk	Low risk
Overall risk of bias	Low risk	Low risk

*ROB 2*, risk of bias 2.

### Statistical analyses

We used structured approaches, including tabulation and narrative description, to organize the evidence. Challenges in performing a meta-analysis, such as variations in data presentation across studies or data being available from only a single study, led us to rely on descriptive methods to report the findings.

## Results

The initial search strategy identified 782 studies. Following title and abstract screening, 13 studies remained for full-text review, and 7 met criteria for final inclusion. Of these, 2 were RCTs (randomized controlled trials), and 5 were retrospective series. In total, 33,159 procedures (anatomic total shoulder arthroplasty, reverse total shoulder arthroplasty, hemiarthroplasty, and humeral head arthroplasties) were included. The study characteristics and baseline demographic information is shown in Table III, with the results of each study summarized in Table IV.

**Table III:** Characteristics of the included studies and patients within the studies.

Study	Level of evidence	Study period	Patients	Mean age (yr)	N (%) male	MRSA colonization (N, % of individual cohorts)	Penicillin/cephalosporin allergy (N, % of individual cohorts)	Intervention	Intervention	Comparator
Peel et al, 2023^24^	I	2019-2021	30 aTSA or rTSA	66.6 ± 10.5	Not reported	MRSA colonized patients not included	Cefazolin and vancomycin allergic patients not included	Vancomycin 1.5 g given within 120 min of incision + cefazolin 2 g given 60 min before incision, N = 15	Cefazolin 2 g given 60 min before incision, N = 15	
Rao et al, 2018^25^	I	2016-2017	29 aTSA 27 rTSA	Doxycycline + cefazolin: 66.9 ± 7.9 Cefazolin: 68.6 ± 8	33 (59%)	Not reported	Penicillin or doxycycline allergic patients not included	Doxycycline: 100 mg IV + cefazolin: weight-based dose (2-3 g); N = 29	Weight-based cefazolin begun 1 h prior to incision and continued 24 h postop, N = 27	
Dettmer et al, 2023^6^	III	January 1, 2013-December 31, 2019	1,696 HA 4,159 aTSA 4,722 rTSA	70 ± 10 Age was not reported by abx group	3,822 (36%)	Not reported	Not reported	Clindamycin, N = 4,264	Cloxacillin + benzylpenicillin, N = 3,709	Cloxacillin, N = 2,604
Marigi et al, 2022^13^	III	2000-2019	877 HA 3,309 aTSA 3,527 rTSA	Cefazolin: 68.6 ± 11.5 Non–cefazolin: 68.7 ± 11.7	3,692 (48%)	Cefazolin: 519 (7.5%) Vancomycin: 57 (12.3%) Clindamycin: 37 (10.7%) *P* < .001	Not reported	Vancomycin, N = 465	Clindamycin, N = 345	Cefazolin, N = 6,879
Marigi et al, 2023^14^	III	2000-2019	47 HA 223 aTSA 191 rTSA	Incomplete: 66.8 ± 12.8 Complete: 69.5 ± 11.4	184 (39.9%)	Complete: 34 (11.4%) Incomplete: 23 (14.1%) *P* = .460	Complete: 282 (94.6%) Incomplete: 158 (96.9%) *P* = .236	Complete vancomycin, infusion time > 30 min; N = 298	Incomplete vancomycin, infusion time < 30 min; N = 163	
Marigi et al, 2024^15^	III	2000-2019	828 HA 3,122 aTSA 3,232 rTSA	Cefazolin: 68.6 ± 11.5 Vancomycin: 69.5 ± 11.4	3,381 (49.2%)	Vancomycin: 34 (11.4%) Cefazolin: 519 (7.5%) *P* = .014	Vancomycin: 282 (94.6%)	Vancomycin, infusion time > 30 min;, N = 298	Cefazolin, 2 grams preop; N = 6,879	
Yian et al, 2020^32^	III	2005-2016	7,140 total (aTSA, rTSA, HA, humeral head arthroplasty)	6,062 (84.9%) were ≥60 year old	3,177 (44.5%)	not reported	Vancomycin group: 444 (100%) Clindamycin group: 508 (100%)	Vancomycin, N = 444	Clindamycin, N = 508	Cefazolin, N = 6,188

*aTSA*, anatomic total shoulder arthroplasty; *rTSA*, reverse total shoulder arthroplasty; *HA*, hemiarthroplasty; *MRSA*, methicillin-resistant *Staphylococcus aureus*.

**Table IV:** Results of included studies.

Study	Level of evidence	Outcome studied	All-cause positive infection results, N (%)			*C. acnes positive results, N (%)*			Methicillin resistant bacteria positive results, N (%)			Mean follow up duration	Adjusted statistics	Antibiotic associated adverse events
			Intervention	Intervention	Comparator	Intervention	Intervention	Comparator	Intervention	Intervention	Comparator			
Peel et al, 2023^24^	I	Superficial, deep, and organ-space infections	Vancomycin + cefazolin: 1 (6.7%)		Cefazolin only: 0 (0%)	Not reported			Not reported			180 d	Not reported	Not reported specifically within shoulder cohort
Rao et al, 2018^25^	I	*C. acnes* positive intraoperative cultures	Not reported			Doxycycline + cefazolin: 11 (38%)		Cefazolin: 10 (37%)	Not reported			Not reported	Not reported	No doxycycline related adverse events
Dettmer et al, 2023^6^	III	PJI requiring reoperation	Cloxacillin and benzylpenicillin: 18 (0.49%)	Clindamycin alone: 33 (0.77%)	Cloxacillin alone: 38 (1.45%)	Not reported			Not reported			989 ± 669 d	HR associated with cloxacillin use: Compared to cloxacillin and benzylpenicillin: 2.40 (1.35-4.25) Compared to clindamycin alone: 1.78 (1.11-2.85) Cloxacillin + benzylpenicillin vs. clindamycin: 0.74 (0.42- 1.32)	Not reported
Marigi et al, 2022^13^	III	PJI	Vancomycin: 11 (2.4%)	Clindamycin: 14 (4.1%)	Cefazolin: 76 (1.1%)	Overall *C. acnes*: 44 (0.57%)			Overall MRSA: 3 (0.04%) Overall MRSE: 1 (0.01%)			5.3 ± 3.6 y	HR when compared to cefazolin Vancomycin: All-cause: 2.32 (1.22-4.4) *C. acnes*: 2.94 (1.12-7.49) Clindamycin: All-cause: 5.07 (2.83-9.05) *C. acnes*: 8.01 (3.63-17.42)	Not reported
Marigi et al, 2023^14^	III	PJI	Vancomycin (infusion > 30 min): 7 (2.3%)		Vancomycin (infusion <30 min): 13 (8.0%)	Vancomycin (infusion > 30 min): 2 (0.7%)		Vancomycin (infusion <30 min): 4 (2.5%)	Not reported			6.5 (range, 2-22) yr	HR when compared to vancomycin (infusion > 30 min) Infusion < 30 min All-cause: 5.26 (1.42-19.52)	Not reported
Marigi et al, 2024^15^	III	PJI	Vancomycin (infusion > 30 min): 3 (1%)		Cefazolin: 76 (1.1%)	Vancomycin (infusion > 30 min): 2 (0.67%)		Cefazolin: 31 (0.45%)	Vancomycin (infusion > 30 min): MRSA: 1 (0.34%) MRSE: 0 (0%)		Cefazolin: MRSA: 1 (0.01%) MRSE: 1 (0.01%)	5.4 (range, 2-22) years	HR when compared to cefazolin Vancomycin > 30 min All-cause: 1.5 (0.7-3.25)	Not reported
Yian et al, 2020^32^	III	Deep infection and/or revision surgery due to infection	Vancomycin: 4 (1.1%)	Clindamycin: 13 (3.2%)	Cefazolin: 53 (1.0%)	Vancomycin: 1 (0.3%)	Clindamycin: 5 (1.3%)	Cefazolin: 21 (0.4%)	Overall MRSA: 2 (0.02%)			5 yr	HR when compared to cefazolin Vancomycin All-cause: 1.17 (0.42-3.30) *C. acnes:* 0.85 (0.15-4.90) Clindamycin All-cause: 3.45 (1.84-6.47) *C. acnes:* 4.04 (1.25-13.06)	Not reported

*HR*, hazard ratio; *C. acnes*, *Cutibacterium acnes*; *MRSA*, methicillin-resistant *Staphylococcus aureus*; *MRSE*, methicillin-resistant *Staphylococcus epidermidis*; *PJI*, periprosthetic joint infection.

These studies allowed us to compare utilization rates and infection prophylactic efficacy of cefazolin compared to non–cefazolin antibiotics.

### Vancomycin and cefazolin

One RCT and 4 retrospective studies compared infection rates between SA patients treated with vancomycin or cefazolin.^13^, ^14^, ^15^^,^^24^^,^^32^ Sample size varied from 30 to 7,713 patients with 15-444 receiving vancomycin and 15-6,879 patients receiving cefazolin. In the RCT of 30 SA patients, 1 (6.7%) surgical site infection occurred in the vancomycin and cefazolin cohort compared to 0 (0%) in the cefazolin-only cohort.^24^ Among the retrospective studies, the all-cause infection rate ranged from 1.0%-2.4% for vancomycin and 1.0%-1.1% for cefazolin-treated patients. One study found an increased risk of PJI associated with vancomycin-only prophylaxis compared to cefazolin.^13^ However, in a follow-up study, the same authors assessed whether duration of antibiotic administration affected infection rates following SA.^14^ In this study, 298 SA patients with “complete” (infusion started > 30 minutes preoperatively) vancomycin dosing were compared to 163 with “incomplete” (infusion started < 30 minutes preoperatively) prophylaxis. The incomplete administration group had a significantly higher rate of all-cause PJIs (5.5% vs. 1%, *P* = .004).^14^ In their third study, the authors found no increased risk of PJI when comparing patients who received complete vancomycin administration at the time of incision to those who received cefazolin.^15^ A separate research group also found no increased risk of all-cause or *C. acnes*–related surgical site infection between patients treated with vancomycin or cefazolin.^32^ Thus, timing of vancomycin administration likely is important in achieving complete prophylaxis.

### Clindamycin and cefazolin

Two retrospective studies with sample sizes of 7,140 and 7,224 compared infection rates between SA patients receiving clindamycin, vancomycin, or cefazolin.^13^^,^^32^ All-cause infection rates ranged from 3.2%-4.1% for clindamycin, 1.1%-2.4% for vancomycin, and 1.0%-1.1% for cefazolin.^13^^,^^32^ In both studies, clindamycin was associated with increased infection risk compared to cefazolin after controlling for confounders such as age, sex, Charlson Comorbidity Index, MRSA colonization, and use of antibiotic cement (Table IV).

### Cefazolin and doxycycline

One RCT that included 56 SA patients compared intraoperative culture positivity rates between patients treated with cefazolin and doxycycline against those treated with cefazolin and placebo. Cultures were sampled from the skin, deep dermal tissue, and glenohumeral joint. No significant difference in the rate of ≥ 1 intraoperative culture was noted between the treatment (n = 11, 39%) and placebo (n = 10, 37%) cohorts (*P* = .99).^25^ When stratifying by culture location, the authors did not find significant differences (Table IV).

### Cloxacillin and clindamycin

One retrospective study analyzed 10,577 SAs from the Swedish Shoulder Arthroplasty Register, evaluating the efficacy of 3 antibiotic prophylaxis regimens: cloxacillin, clindamycin, and a combination of benzylpenicillin and cloxacillin. Cloxacillin monotherapy was associated with a significantly higher risk of reoperation for infection compared to the benzylpenicillin–cloxacillin combination (hazard ratio [HR] 2.40; *P* = .003) and clindamycin alone (HR 1.78; *P* = .02). No significant difference was found between clindamycin and the regimen of benzylpenicillin and cloxacillin (HR 0.74; *P* = .31).^6^

## Discussion

A review of the literature indicates that cefazolin is the most commonly used prophylactic antibiotic for sSAs. Alternatives to cefazolin are primarily considered for patients with a reported penicillin allergy or, less frequently, for those with methicillin-resistant bacterial colonization. Among non–cefazolin antibiotics, vancomycin appears more effective than clindamycin when fully administered, while evidence supporting doxycycline or cloxacillin/benzylpenicillin use remains limited. Overall, this review supports cefazolin as the preferred prophylactic option and underscores the need for an evidence-based decision-making algorithm for SA patients with penicillin allergies or methicillin-resistant bacterial colonization.

The present data suggests that vancomycin is likely superior to clindamycin for prevention of PJI in patients who cannot receive cefazolin due to a true penicillin allergy. One potential explanation is increasing *C. acnes* resistance to clindamycin, which has been reported in almost 22% of *C. acnes* isolates.^5^^,^^20^ For example, using *in vitro* and foreign-body infection models, Tafin et al reported a minimum bactericidal concentration of 512 μg/ml for clindamycin (compared to 8 μg/ml for vancomycin) when treating *C. acnes*.^8^ However, as these data are derived from retrospective or nonclinical works, a cautious appraisal is warranted, and high-quality randomized trials are needed to make definitive recommendations. Of note, the combined use of vancomycin and clindamycin has also gained traction among SA surgeons, though the improved bacterial coverage afforded by this practice must be balanced with the risk of antibiotic-related adverse events and proper antibiotic stewardship.^2^^,^^22^^,^^23^

Despite its well-documented efficacy against *C. acnes* in acne vulgaris*,* doxycycline has not proved as useful for SA prophylaxis.^19^^,^^25^ The current study did not find a significant reduction in intraoperative *C. acnes* culture positivity rates among SA patients receiving doxycycline.^25^ Similarly, a randomized trial by Namdari et al reported that a 7-day course of oral doxycycline before shoulder arthroscopy did not significantly decrease *C. acnes* colonization compared to no treatment.^19^ The authors hypothesized that this may be related to the drug's bacteriostatic mechanism rather than directly eliminating the bacteria.^25^ As such, despite its overall safety profile and efficacy for treating MRSA, there is limited evidence supporting its use as an adjunct for SA infection prevention. Future investigations should explore the clinical relevance of the aforementioned positive intraoperative cultures and whether the addition of doxycycline will lead to meaningful reductions in postoperative infection rates.

Interpreted within the appropriate context, this review supports cefazolin as the primary prophylactic agent for SA and recommends its use whenever possible. Patients with methicillin-resistant bacterial colonization where vancomycin is indicated may benefit from the addition of cefazolin due to improved coverage and potential synergistic antibacterial effects.^3^^,^^4^ For patients reporting a penicillin allergy, preoperative evaluation of the allergy severity is critical as 95% of reported allergies are not true immunoglobulin E-mediated reactions.^27^^,^^30^ In addition, the cross-reactivity between penicillin and first-generation cephalosporin is estimated to occur at a rate of 0.7%.^33^ As such, those with no or mild reported reactions should receive cefazolin, while those with unknown reactions may perform outpatient or perioperative testing.^16^ If the test is well tolerated, cefazolin should be used; otherwise, or in cases of severe reactions (eg, toxic epidermal necrolysis or Stevens-Johnson Syndrome), an alternative antibiotic should be administered.^26^

This systematic review has several limitations that warrant consideration. First, the data on antibiotic prophylaxis in the SA cohort is predominantly derived from retrospective studies, which introduces inherent biases to patient selection and data procurement. In addition, there is heterogeneity among the included studies, particularly in terms of patient populations and the antibiotic regimens utilized, making direct comparisons challenging. However, the consensus across multiple studies lends credibility to their findings. Despite these limitations, this review offers valuable insights into the contemporary landscape of antibiotic prophylaxis in SA. Notably, the study provides a comprehensive synthesis of the existing literature, highlighting trends in antibiotic usage and identifying knowledge gaps that warrant further investigation.

## Conclusion

This systematic review highlights that cefazolin should be used as frequently as possible for prevention of SA infection. Current evidence suggests that fully administered vancomycin is a superior non–cefazolin alternative compared to clindamycin. Current evidence does not support the use of doxycycline. Future research should prioritize incorporating allergy testing to better identify patients who genuinely require non–cefazolin antibiotics, continue to utilize robust methodologies, and explore the potential benefits of dual antibiotic regimens.

## Disclaimers

Funding: No funding was disclosed by the authors.

Conflicts of interest: Brian Feeley receives funding from an R01 grant (R01AR072669), holds stock options with Bioniks and Kaliber.ai, and is on the editorial board of the Journal of Shoulder and Elbow Surgery. All the other authors, their immediate families, and any research foundations with which they are affiliated have not received any financial payments or other benefits from any commercial entity related to the subject of this article.
