# Supplementary material for: Preoperative antibiotic prophylaxis in primary shoulder arthroplasty patients: a systematic review
Source: JSES Int. 2025 Jul 7;9(6):2062–8. doi: 10.1016/j.jseint.2025.06.010 (PMC12828191; doi:10.1016/j.jseint.2025.06.010)
Supplement: Appendix [file mmc1.docx]

Appendix

Title and Abstract Search Strategy for Antibiotic Prophylaxis in Shoulder Arthroplasty Patients

Search date: 9/25/2024

PubMed:

("Arthroplasty, Replacement, Shoulder"[Mesh] OR "shoulder arthroplasty" OR "shoulder replacement" OR hemiarthroplasty OR "shoulder replacement arthroplasty" OR (arthroplasty AND ("Shoulder/surgery"[Mesh] OR “shoulder surgery”))) AND (preoperative OR "Preoperative Care"[Mesh] OR "Preoperative Period"[Mesh] OR prophylactic OR prophylaxis OR "Antibiotic Prophylaxis"[Mesh] OR premedication OR "Premedication"[Mesh])

AND (vancomycin OR "Vancomycin"[Mesh] OR ancef OR cefazolin OR "Cefazolin"[Mesh] OR kefzol OR clindamycin OR "Clindamycin"[Mesh] OR cleocin OR doxycycline OR "Doxycycline"[Mesh] OR doryx OR vibramycin OR antimicrobial OR antibiotic OR antibiotics OR antibacterial OR "Anti-Bacterial Agents"[Mesh])

Web of Science:

("shoulder arthroplasty" OR "shoulder replacement" OR hemiarthroplasty OR "shoulder replacement arthroplasty" OR (arthroplasty AND “shoulder surgery”)) AND (preoperative OR prophylactic OR prophylaxis OR premedication) AND (vancomycin OR ancef OR cefazolin OR kefzol OR clindamycin OR cleocin OR doxycycline OR doryx OR vibramycin OR antimicrobial OR antibiotic OR antibiotics OR antibacterial)

Embase:

('shoulder arthroplasty'/exp OR 'shoulder arthroplasty' OR 'shoulder replacement'/exp OR 'shoulder replacement' OR 'hemiarthroplasty'/exp OR hemiarthroplasty OR 'shoulder replacement arthroplasty'/exp OR 'shoulder replacement arthroplasty' OR (('arthroplasty'/exp OR arthroplasty) AND ('shoulder surgery'/exp OR 'shoulder surgery'))) AND (preoperative OR prophylactic OR 'prophylaxis'/exp OR prophylaxis OR 'premedication'/exp OR premedication) AND ('vancomycin'/exp OR vancomycin OR 'ancef'/exp OR ancef OR 'cefazolin'/exp OR cefazolin OR 'kefzol'/exp OR kefzol OR 'clindamycin'/exp OR clindamycin OR 'cleocin'/exp OR cleocin OR 'doxycycline'/exp OR doxycycline OR 'doryx'/exp OR doryx OR 'vibramycin'/exp OR vibramycin OR 'antimicrobial'/exp OR antimicrobial OR 'antibiotic'/exp OR antibiotic OR 'antibiotics'/exp OR antibiotics OR 'antibacterial'/exp OR antibacterial) AND ('article'/it OR 'article in press'/it)

References = 402

Cochrane (CENTRAL):

("shoulder arthroplasty" OR "shoulder replacement" OR hemiarthroplasty OR "shoulder replacement arthroplasty" OR (arthroplasty AND “shoulder surgery”)) AND (preoperative OR prophylactic OR prophylaxis OR premedication) AND (vancomycin OR ancef OR cefazolin OR kefzol OR clindamycin OR cleocin OR doxycycline OR doryx OR vibramycin OR antimicrobial OR antibiotic OR antibiotics OR antibacterial)

CINAHL Complete:

("shoulder arthroplasty" OR "shoulder replacement" OR hemiarthroplasty OR "shoulder replacement arthroplasty" OR (arthroplasty AND “shoulder surgery”)) AND (preoperative OR prophylactic OR prophylaxis OR premedication) AND (vancomycin OR ancef OR cefazolin OR kefzol OR clindamycin OR cleocin OR doxycycline OR doryx OR vibramycin OR antimicrobial OR antibiotic OR antibiotics OR antibacterial)
